# Seroprevalence of human hantavirus infection in the Ribeirão Preto region of São Paulo State, Brazil.

**DOI:** 10.3201/eid0605.000523

**Published:** 2000

**Authors:** R Holmes, R Boccanera, L T Figueiredo, S R Mançano, C Pane


7. Nolte KB, Simpson GL, Parrish RG. Emerging infectious agents and the forensic pathologist: the New Mexico model. Arch Pathol Lab Med 1996;120:125-8.
8. Walker DH, Yampolska O, Grinberg LM. Death at Sverdlovsk: what have we learned? Am J Pathol 1994;144:1135-41.
9. Lifschultz BD, Donoghue ER. The Tylenol cyanide poisonings. American Academy of Forensic Sciences 1989;46(Abstract A12).

### Seroprevalence of Human Hantavirus Infection in the Ribeirão Preto Region of São Paulo State, Brazil

**To the Editor:** Hantavirus pulmonary syndrome (HPS) has been identified in the region of Ribeirão Preto, São Paulo State, Brazil, since 1993 (1-4). As of September 1999, 38 HPS cases had been reported in Brazil, 16 in the state of São Paulo (2). Between May 1998 and August 1999, the Adolfo Lutz Institute (ALI) in São Paulo city serologically confirmed five cases—three fatal—in the Ribeirão Preto region: two from Guariba, one from Jardinópolis, one from Cajuru, and one from Cassia dos Coqueiros (Luiza Teresinha Madia de Souza, ALI, pers. comm.).

Despite these reports and suspicions of additional cases, the prevalence of hantavirus infection and HPS in the region is not known. Laboratory confirmation has not been available locally, and sending serum samples to ALI for laboratory evaluation is not feasible in most cases. Thus, only presumptive diagnoses could be made until the Sin Nombre virus (SNV) enzyme-linked immunosorbent assay (ELISA) was developed.

To estimate the occurrence and distribution of human hantavirus infection, we used SNV ELISA to conduct a serologic survey of a sample of hospital patients requiring venipuncture for routine procedures. The patients came from three regional cities: Ribeirão Preto, Guariba, and Jardinópolis. Between February and June 1999, a total of 567 samples were collected: 257 from the public hospital of Guariba, 110 from the public hospital in Jardinópolis, and 200 from the General Hospital of the School of Medicine of Ribeirão Preto. When we compared our sample with the general population, the patients in the study sample were slightly older but similar in sex distribution.

Sixteen additional samples were evaluated to confirm the effectiveness of SNV ELISA in diagnosing hantavirus infection: 12 from patients in whom HPS was clinically suspected and 4 previously confirmed by ALI in the city of São Paulo between May 1998 and August 1999. Known HPS convalescent-phase plasma provided by the Centers for Disease Control and Prevention (CDC) was used as positive control. Negative controls were selected by simple random sampling from all previously negative samples.

Positive and negative recombinant SNV antigens provided by CDC were coated on microtiter plates at 1:2,000 dilution in phosphate-buffered saline overnight at 4°C and used in a standard immunoglobulin G testing format. Reverse transcriptase-polymerase chain reaction analysis of serum from two fatal cases of HPS occurring in the cities of Franca and Araraquara suggested the presence of two genetically distinct hantaviruses in the area surrounding Ribeirão Preto. Antigen prepared from local virus is not considered to be necessary for immunoassays because the local virus is not sufficiently different from other isolates to require special antigen preparation (5).

All samples were screened in duplicate on both positive and negative antigens in the assay. A sample was considered positive if absorbance on the positive antigen was greater than absorbance on both the negative control antigen and the negative control of the plate. To confirm the diagnosis, samples satisfying these criteria were tested in duplicate along with 14 negative samples. Samples were considered positive when their subtractive absorbance was greater than the calculated mean subtractive absorbance of the 14 negative samples and three standard deviations.

From our serologic survey, the seroprevalence of human hantavirus infection was determined to be 1.23% (7/567) overall, 0.5% (1/200) in Ribeirão Preto, 0.4% (1/257) in Guariba, and 4.5% (5/110) in Jardinópolis. If one assumes the inhabitants sampled were representative, the seroprevalence provides an estimate of surviving past or recent hantavirus infections in the area. As the overall antibody prevalence of 1.23% is more than twice that observed in the U.S. populations at risk for hantavirus infection, such infections are not rare in the Ribeirão Preto region (6).

Three of the four HPS samples previously confirmed by the ALI in São Paulo tested positive by our ELISA. Of the remaining 12 suspected HPS cases assayed, three were positive. Two of these three were later confirmed as positive by the ALI (Luiza Teresinha Madia de Souza, ALI, pers. comm.) Thus, we report three previously unconfirmed HPS cases, one fatal, in the Ribeirão Preto area between May 1998 and August 1999.

Since the rodent reservoir is not known and the virus has not been isolated, rodent capture is currently being conducted in areas where human infections have been found. In addition, positive cases are being retrospectively investigated.

Thanks to Thomas Ksiazek for providing recombinant SNV antigen and positive serum and to Robert Shope for his assistance.

The study was partially funded by grants from the National Institutes of Health, Bethesda, Maryland, USA, and Fundação de Amparo à Pesquisa do Estado de São Paulo, Brazil.

**Ryan Holmes,* Rodrigo Boccanera,†**
**Luiz Tadeu M. Figueiredo,†**
**Silvia Regina Mançano,‡ Clodoaldo Pane§**
*University of Texas Medical Branch at Galveston, University of Texas School of Public Health at Houston, Texas, USA; †Unidade Multidepartamental de Pesquisa em Virologia da Faculdade de Medicina de Ribeirão Preto-USP, Brazil; ‡Centro de Saúde de Guariba, São Paulo State, Brazil; and §Centro de Saúde de Jardinópolis, São Paulo State, Brazil.

### References

1. Figueiredo LTM, Moreli ML, Almeida VSO, Félix PR, Bruno, JC, Ferreira IB, et al. Hantavirus pulmonary syndrome (HPS) in Guariba, SP, Brazil. A report of 2 cases. Rev Inst Med Trop Sao Paulo 1999; 41:131-7.
2. Johnson AM, de Souza LTM, Ferreira IB, Pereira LE, Ksiazek TG, Rollin PE, et al. Genetic investigation of novel hantaviruses causing fatal HPS in Brazil. J Med Virol 1999;59:527-35.
3. Ferreira MS, Nishioka SA, Santos TL, Santos RP, Santos PS, Rocha A. Hantavirus pulmonary syndrome in Brazil: clinical aspects of three new cases. Rev Inst Med Trop Sao Paulo 2000;42:41-6.
4. Da Silva MV, Vasconcelos MJ, Hidalgo NT, Veiga AP, Canzian M, Marotto PC, et al. Hantavirus pulmonary syndrome: report of the first three cases in São Paulo, Brazil. Rev Inst Med Trop Sao Paulo 1997;39:231-4.
5. Peters CJ. Emerging infections: hantavirus pulmonary syndrome in the Americas. Washington: ASM Press;1998.
6. Zeitz PS, Butler JC, Cheek JE, Samuel MC, Childs JE, Shands LA, et al. A case-control study of hantavirus pulmonary syndrome during an outbreak in the southwestern United States. J Infect Dis 1995;171:864-70.

### Molecular Characterization of *Mycobacterium abscessus* Strains Isolated from a Hospital Outbreak

**To the Editor:** In recent years there have been several reports of sporadic and epidemic hospital-acquired infections caused by rapidly growing mycobacteria, namely, *Mycobacterium abscessus*, *M. chelonae*, and *M. fortuitum*. *M. abscessus* has been well documented as a cause of cutaneous and soft tissue infections and has been implicated in chronic ear infections, bacteremia associated with hemodialysis equipment, and peritoneal dialysis–related infections (1).

Differentiating mycobacteria to the species level is difficult because of the diversity of available techniques and the time required for full identification. A rapid method based on the evaluation of the gene coding for the 65-kDa heat shock protein, which contains epitopes both unique and common to various species of mycobacteria, has been reported (2). A 383-bp sequence situated at the amino terminus of this 65-kDa antigen (3) has been shown to be conserved among several species of mycobacteria. Reports based on polymerase chain reaction (PCR) amplification and DNA sequencing (4) show species-specific polymorphism at the nucleotide level within this region (5). The conserved nature of this gene allows differentiation of mycobacteria within 1 day by restriction enzyme digestion of PCR products obtained by using primers common to all mycobacteria.

From August through December 1995, postoperative wound infections developed in 45 patients in the pediatric surgery unit of Kalawati Saran Children’s Hospital, New Delhi, India; 42 were day-care patients, and 3 were inpatients who had undergone major surgery. Thirty-two clinical samples (pus and exudate) were tested for acid-fast bacilli by the Ziehl-Neelson method; the same smear samples were cultured on Lowenstein-Jensen slants and examined for growth daily for 4 days and thereafter twice a week for 8 weeks. The

